# The Role of *Chlorella* and Spirulina as Adjuvants of Cardiovascular Risk Factor Control: A Systematic Review and Meta-Analysis of Randomised Controlled Trials

**DOI:** 10.3390/nu17060943

**Published:** 2025-03-07

**Authors:** Mariana Pinto-Leite, Diana Martins, António Carlos Ferreira, Cláudia Silva, Fábio Trindade, Francisca Saraiva, Rui Vitorino, Raquel Barros, Pedro A. Lima, Adelino Leite-Moreira, João Pedro Ferreira, António S. Barros, Isabel M. Miranda

**Affiliations:** 1 RISE-Health, Department of Surgery and Physiology, Faculty of Medicine, University of Porto, 4200-319 Porto, Portugal; 2 iBiMED, Department of Medical Sciences, University of Aveiro, 3810-193 Aveiro, Portugal; 3 Sea4Us, SA, Porto da Baleeira, Armazém 8, 8650-368 Sagres, Portugal

**Keywords:** algae, *Chlorella*, Spirulina, cholesterol, triglycerides, blood pressure, cardiometabolic diseases, meta-analysis

## Abstract

**Background/Objectives**: *Chlorella* and Spirulina supplementation may reduce the risk of cardiometabolic diseases by better controlling blood cholesterol, triglycerides, glucose, weight, and blood pressure (BP). However, the available studies are limited in size and have used different outcomes. **Methods**: To gain power in assessing the impact of microalgae supplements on cardiovascular risk factors, we searched PubMed on 3 February 2023 for randomised controlled trials assessing the effects of *Chlorella* and Spirulina on modifiable cardiovascular risk factors. **Results**: We found 12 studies in *Chlorella* and 9 studies on Spirulina. Depending on the available outcomes, varying numbers of participants (*Chlorella*: 168 to 279; Spirulina: 101 to 299) were included. Our analysis showed that *Chlorella* supplementation had a neutral effect on BP and lipemia. On the other hand, Spirulina intake led to a significant reduction in diastolic BP (−0.42, 95% CI: −0.81 to −0.02, *p* = 0.04) but did not significantly affect lipemia indexes, despite a trend toward a reduction in total cholesterol (−0.17, 95% CI: −0.39 to 0.06, *p* = 0.15). This meta-analysis suggests Spirulina supplementation can be used as an adjuvant to control cardiometabolic risk factors, particularly for BP. However, the magnitude of this effect is small and of uncertain clinical significance. **Conclusions**: Further randomised trials are needed to better assess the potential of these supplements as adjuvants for the control of cardiovascular risk factors.

## 1. Introduction

Although the consumption of algae has been prevalent among Asian populations, such as Koreans and Japanese people, since ancient times, it was only in the 15th century that it was introduced into European gastronomy [1]. Algae is a taxonomically diverse group of species, comprising all macroalgae (also known as seaweed), a large and diverse group of photosynthetic eukaryotic organisms, and microalgae, including eukaryotic algae and some prokaryotic cyanobacteria, which often appear in multicellular forms [2].

Macroalgae, which can be subdivided into red, brown, and green algae, are highly nutritious and contain relatively high amounts of proteins and low amounts of fat [3]. However, the availability of different types of algae for consumption varies among countries depending on specific microbiological criteria and regulations. Nevertheless, there is unanimous interest in algal biomass as a source of soluble non-digestible polysaccharides, such as alginate, charide, fucoidan, carrageenan, and exopolysaccharides, owing to their prebiotic effects [1]. Polysaccharide composition varies among the different algal divisions. Brown algae comprise alginate, laminarin, and fucoidan, while red algae contain agar and carrageenan, and green algae comprise ulvan [3].

Microalgae, as the name suggests, are microscopic organisms with a short generation time that can be found in fresh water and seawater [1]. These are the primary sources of carotenoids, a group of molecules known for their antioxidant properties, making them highly commercially valuable [4]. β-carotene, the most abundant carotenoid in the human diet, is particularly noteworthy [5]. Astaxanthin also has remarkable antioxidant properties, protecting the inner and outer cell membrane layers against oxidative stress. This carotenoid has already been approved as a dietary supplement and has shown potential to protect β-cells from glucose toxicity, as observed in diabetic mice [6]. Cyanobacteria, specifically Spirulina, have gained considerable attention as prokaryotic microalgae, mainly because of their utilisation as dietary supplements by the National Aeronautics and Space Administration (NASA) for astronauts [7]. Other cyanobacteria, such as *Nostoc*, *Anabaena*, and *Oscillatoria*, have also been extensively studied for their potential as effective anticancer drugs [8]. This is attributed to cyanobacterial peptides. Their ability to produce diverse secondary metabolites makes them promising candidates for combating resistant pathogens and emerging diseases [9]. Spirulina (*Limnospira* sp.) and *Chlorella* (*Chlorella vulgaris*), two microalgae that have been widely researched, were selected as the focus of this systematic review due to their global commercial availability, established safety profiles, and the existence of sufficient randomised controlled trials (RCTs) to enable meaningful meta-analysis. According to previous research, these microalgae may improve immune function and reduce inflammation. Research has concentrated on their influence on lipid profiles and fluctuations in body composition parameters [8].

The latest edition of the World Health Statistics published by the World Health Organization (WHO) still considers non-communicable diseases (NCDs) to have the highest disease burden worldwide [10]. It emphasises four NCDs, namely, cancer, chronic respiratory disease, diabetes, and cardiovascular diseases (CVDs), which jointly resulted in approximately 33 million fatalities globally in 2019. Despite a 27% reduction in CVD incidence from 2000 to 2019, these ailments continue to cause almost 18 million deaths annually. In contrast, the incidence of diabetes has increased by 3%, leading to 2 million fatalities. Behavioural factors, including excessive alcohol consumption, smoking, unhealthy diet, and lack of physical activity, significantly contribute to the metabolic risk factors associated with these conditions, such as hypertension, hyperglycaemia, hyperlipidaemia, and overweight/obesity. In 2019, the probability of dying from NCDs between the ages of 30 and 70 years was 17.8% [10]. However, there is a strong likelihood that this number will continue to increase. Therefore, exploring new strategies to address this issue is of utmost importance.

The primary aim of this systematic review was to thoroughly evaluate the existing evidence on the potential positive effects of *Chlorella* and Spirulina supplementation on the control of risk factors associated with NCDs, with a focus on cardiometabolic health.

## 2. Materials and Methods

This systematic review was designed according to the 2020 updated guidelines for systematic reviews provided by the Preferred Reporting Items for Systematic Reviews and Meta-Analyses (PRISMA) [11]. This meta-analysis was registered in the Open Science Framework (OSF) [12].

### 2.1. Article Retrieval

Articles were retrieved from the PubMed database. On 3 February 2023, 369 articles were screened using the following query: algae OR alga OR seaweed OR spirulina OR laminara OR chlorella OR gelidium. The search was limited to RCTs and was complemented by manually reviewing the reference lists of all retrieved articles to ensure comprehensive coverage of the relevant literature.

Initially, we searched for studies on multiple algae species, including *Laminara* and *Gelidium*, to comprehensively assess algae’s effects on cardiovascular risk factors. However, the limited availability of high-quality RCTs for these species prevented meaningful meta-analysis. Therefore, we narrowed the scope of the study to focus on Spirulina and *Chlorella*, which have sufficient clinical evidence to support statistical analysis. To streamline and expedite the screening and selection of appropriate studies, the web tool “Rayyan” [13] was used, and data were analysed using the Review Manager Web software (RevMan) [14].

### 2.2. Study Selection

Studies that fulfilled the following criteria were included: human population (healthy or with metabolic disease) and available baseline and post-intervention evaluations (follow-up). Articles that did not meet these criteria and/or those that reported that the study population was taking additional medications, such as antihypertensive medications, were excluded. Articles were first screened by one reviewer, with reference lists of potentially included articles rechecked by two additional reviewers.

### 2.3. Data Collection Process and Items

For both the Spirulina and *Chlorella* studies, the publication year, country, dosage, number of subjects in the placebo and intervention groups, age, sex, and intervention duration were independently collected by three reviewers.

Initially, data related to the morphogenic and biochemical outcomes were recorded in Excel. However, only the outcomes supported by at least three articles were considered relevant. Thus, the following outcomes for Spirulina were noted: systolic blood pressure (SBP) and diastolic blood pressure (DBP), total cholesterol (TC), triglycerides (TGs), high-density lipoprotein cholesterol (HDL-C), and low-density lipoprotein cholesterol (LDL-C). For *Chlorella*, the following biochemical parameters were considered: TC, TGs, HDL-C, LDL-C, SBP, and DBP.

### 2.4. Statistical Analysis

All results are presented as the mean ± standard deviation (SD). Data reported as median, minimum, and maximum values (range) and/or the first and third quartiles were converted into means with standard deviations using the approach described by Wan et al. [15]. Mean values obtained after supplementation were normalised with the pre-supplementation values to calculate the standardised mean differences (SMDs) and their corresponding 95% confidence intervals (CIs) using a random-effects model. The following figures of merit were obtained: Tau^2^, Chi^2^, I^2^, and *p*-values. Statistical significance was set at *p* < 0.05. Additionally, a Z-test was performed to test the overall effect size and facilitate comparisons between studies. The results are presented as forest plots.

### 2.5. Risk of Bias Assessment

The quality assessment tool provided by the National Heart, Lung, and Blood Institute (NHLBI) for Controlled Intervention Studies was used [16] to assess the risk of bias in the included studies. An evaluator assessed the potential for bias in the articles featuring *Chlorella* and Spirulina. Following this, two further reviewers were designated independently, one of whom appraised articles on *Chlorella* supplementation, whereas the other assessed studies related to Spirulina intake. Any inconsistencies were settled through consultation with the reviewers.

## 3. Results

### 3.1. Study Identification and Selection

**Figure 1:**
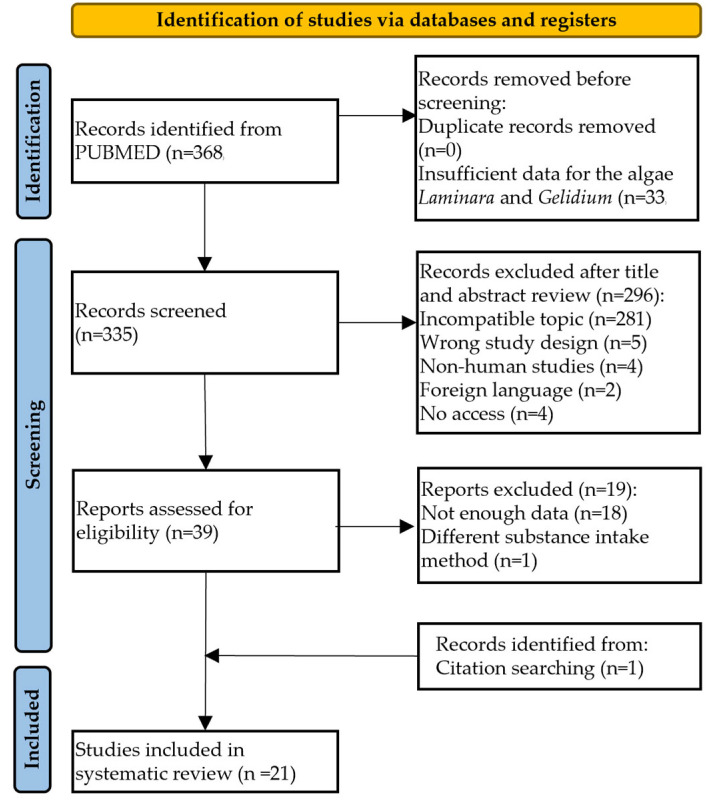
Flow diagram of the study selection procedure.

A total of 368 studies were sourced from the PubMed database using a predefined query string. However, 33 articles were excluded because of insufficient data related to the algae *Laminara* and *Gelidium*. Of the remaining 335 studies, 296 were eliminated based on their titles and/or abstracts during the screening. The exclusion criteria were as follows: incorrect topic (n = 281), inappropriate study design (n = 5), nonhuman studies (n = 4), foreign language (n = 2), and lack of access (n = 4). Thirty-nine articles were subjected to further evaluation; however, eighteen were discarded due to insufficient data, wherein the baseline and post-treatment values were not provided, or information regarding the biochemical parameters and anthropometric measures was unavailable. Additionally, one study was excluded because it used an alternative method of substance ingestion (salad dressing). A total of 21 articles [17,18,19,20,21,22,23,24,25,26,27,28,29,30,31,32,33,34,35,36,37] were selected for the systematic review after conducting a cross-reference analysis, as shown in Figure 1.

### 3.2. Characteristics of the Included Studies

None of the studies on *Chlorella* or Spirulina intake reported adverse effects throughout the trial period. Table 1 (*Chlorella* supplementation studies) and Table 2 (Spirulina supplementation studies) show an overview of the demographic characteristics, clinical tests, and body composition parameters reported in each study.

#### 3.2.1. Chlorella Intake Studies

The *Chlorella* analysis included 12 articles published between 2010 and 2022. The age of the participants varied from 20 to 58 years, and the studies were conducted in Iran (n = 3) (23, 26, 27), South Korea (n = 4) (16, 18, 21, 22), Japan (n = 4) (17, 19, 20), and Taiwan (n = 1) (25). The duration of the trials varied between 4 and 12 weeks, and the *Chlorella* dosage ranged from 1500 to 8000 mg/day.

**Figure 2:**
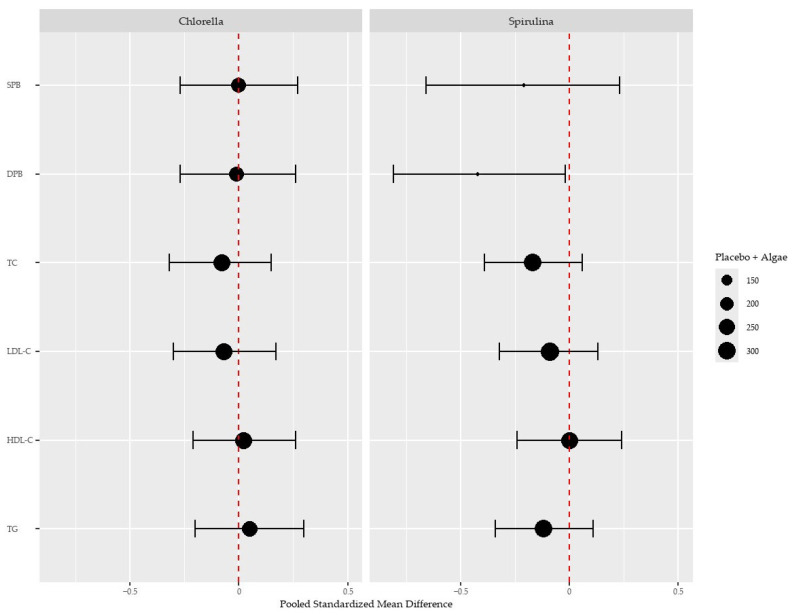
Effects of *Chlorella* and Spirulina treatment over the placebo on systolic (SPB) and diastolic (DPB) blood pressure, total cholesterol (TC), high-density lipoprotein cholesterol (HDL-C), low-density lipoprotein cholesterol (LDL-C), and triglycerides (TGs). The size of each dot represents the sample size. Vertical red lines denote the absence of an effect (standardised mean difference of 0). A leftward shift of the dots to the vertical lines, except for HDL-C, indicates a positive health effect.

Detailed forest plots for the effect of *Chlorella* intake over placebo are provided in the Supplementary Materials (Section S1). We found no effect of *Chlorella* over placebo in any biochemical or blood pressure parameters measured when pooling the available evidence (Figure 2).

Although statistical significance testing of baseline characteristics in RCTs is not recommended to assess the balance between groups, systematic evaluation of these characteristics remains important for understanding the effectiveness of randomisation and identifying potential confounders. Our analysis of the baseline characteristics revealed no imbalances that would suggest compromised randomisation.

#### 3.2.2. Spirulina Intake Studies

A total of nine articles published between 2008 and 2022 were included, with the ages of the participants ranging from an average of 34 to 66 years. The studies were conducted in Iran (n = 3) [34,35,37] and South Korea (n = 2) [29,30], and the rest were conducted in the USA (n = 1) [31], France (n = 1) [36], and Poland (n = 2) [32,33]. The duration of the trials varied, extending from 8 to 16 weeks, as did the dosage of Spirulina supplementation, which ranged from 200 mg/day (liquid extract) to 8000 mg/day.

Detailed forest plots for the effect of Spirulina intake over placebo are provided in the Supplementary Materials (Section S2). Spirulina intake significantly reduced diastolic BP (−0.42, 95% CI: −0.81 to −0.02, *p* = 0.04) but not systolic BP (−0.21, 95% CI: −0.66 to 0.23, *p* = 0.07). The pooled analysis also showed no significant effect on cholesterol and triglycerides despite a trend of a reduction in total cholesterol (−0.17, 95% CI: −0.39 to 0.06, *p* = 0.15) (Figure 2).

### 3.3. Risk of Bias

Based on the National Heart, Lung, and Blood Institute (NHLBI) assessment tool “Quality Assessment of Controlled Intervention Studies” [16], all articles scored an overall “good” risk of bias. Detailed risk of bias assessment scores for each article are provided in the Supplementary Materials and Sections S1 and S2 for *Chlorella* and Spirulina, respectively.

## 4. Discussion

Hypertension and dyslipidaemia are among the crucial factors that contribute to the prevalence of NCDs and pose considerable challenges to global public health. As the incidence of NCDs is linked to increased morbidity and mortality rates, it is essential to investigate complementary approaches to manage these risk factors. This systematic review aimed to evaluate the influence of microalgae supplementation on controlling risk factors associated with cardiometabolic health to evaluate their potential role in primary intervention.

None of the baseline evaluations in the *Chlorella* and Spirulina studies showed differences between the placebo and treated groups, indicating no initial bias.

The consumption of Spirulina significantly reduced diastolic BP, whereas *Chlorella* intake had no effect over placebo. Looking first at the impact of the consumption of algae on BP control, the average baseline BP for the subjects who consumed Spirulina was 135/85 mmHg, and for the subjects who consumed *Chlorella*, the average was 125/78 mmHg. According to the latest 2023 European Society of Cardiology (ESC) guidelines [38], to be in the “Optimal” category, the systolic BP must be less than 120 mmHg, and the diastolic BP must be less than 80 mmHg. This places the Spirulina group in the “High Normal” category and the *Chlorella* group in the “Normal” category. While statistically significant, the observed reduction in diastolic BP with Spirulina (−0.42) is modest and would likely have limited clinical impact when solely considered. However, in multimodal cardiovascular risk management, even small improvements across multiple parameters may contribute to cumulative risk reduction when combined with other dietary and lifestyle interventions. It is worth noting that many nutritional interventions for blood pressure produce similarly modest effects that may become clinically meaningful when part of a comprehensive approach to cardiovascular health. After Spirulina intake, there was a decrease in diastolic BP, placing the trialled individuals closer to the “Optimal” category. The *Chlorella* trials were conducted on individuals who, on average, had a BP reading close to the normal range. This fact might explain the lack of an effect of this alga. Therefore, it would be relevant to test the effect of *Chlorella* consumption on a population with poorer control of BP or hypertension once antihypertensive effects were previously described [39].

Bioactive peptides may explain the BP-lowering effect of Spirulina. An in vivo study showed that Spirulina contains angiotensin-I-converting enzyme (ACE) inhibitory peptides that can inhibit the renin–angiotensin system and, therefore, reduce vasoconstriction and sodium reabsorption, leading to a decrease in BP [40].

In 2021, a comprehensive review of the possible molecular mechanisms of microalgae in ACE provided further evidence and suggested that these microorganisms also possess anti-inflammatory and antioxidant properties. The association between inflammation and oxidative stress, both linked to endothelial dysfunction, reinforces the notion that microalgae can be beneficial for reducing BP [41]. Another study also showed that Spirulina extract can induce endothelial nitric oxide production, leading to further vasodilation and decreased BP [42].

The latest ESC guidelines emphasise the importance of phytosterol intake in patients with dyslipidaemia [43]. Daily phytosterol (2 g) consumption can effectively decrease TC and LDL-C levels by competing with cholesterol for absorption in the gastrointestinal tract. For example, a 5% decline in plasma cholesterol concentration was observed with the intake of 400 mg of phytosterols, which increased to 35–40% when the dose was increased to 1500–2000 mg [44]. Of the many natural phytosterols, *Chlorella* predominantly contains ergosterol, 7-dehydroporiferasterol, ergosterol peroxide, and 7-oxocholesterol [45]. Spirulina, in turn, contains 5.39 ± 2.29% β-sitosterol and 7.61 ± 2.01% stigmasterol, as assessed by thin-layer chromatography [46]. Despite the high content of phytosterols, our meta-analysis showed that neither *Chlorella* nor Spirulina reduced cholesterol levels in algae consumers compared to placebo, despite a trend toward a reduction in total cholesterol in Spirulina. Therefore, additional research is necessary to establish optimal dosages, exposure times, and study sizes to ascertain the true impact of phytosterol-rich algae on plasma cholesterol and triglycerides.

This systematic review has some limitations. First, the available literature on the subject was scarce, and the included articles exhibited considerable heterogeneity in methodology. Intervention dosages varied widely (from 200 mg to 8000 mg daily for Spirulina and 900 mg to 8000 mg for *Chlorella*), as did treatment durations (ranging from 4 to 16 weeks). Furthermore, the study population was diverse, including healthy individuals and patients with various metabolic conditions such as diabetes, obesity, and hypercholesterolemia, which may have influenced response to supplementation. The sample size of most of the studies was relatively small, limiting statistical power and generalisability. This heterogeneity in study parameters may have contributed to the modest effects observed and potentially masked subgroup-specific benefits that could be revealed in a more standardised research protocol. Additionally, other biochemical parameters, such as total antioxidant concentration and Haemoglobin A1C, were not analysed because insufficient articles covering these parameters (less than three) limited the evaluation of significant effects.

## 5. Conclusions

This study demonstrated Spirulina microalgae’s promise as a therapeutic adjuvant for blood pressure management, with a modest but significant reduction in diastolic blood pressure. The magnitude of this effect (−0.42 mmHg), while small, suggests potential utility as a part of a comprehensive approach to cardiovascular risk management rather than as a standalone intervention. Despite theoretical benefits, *Chlorella* supplementation showed neutral effects on the cardiovascular parameters measured in our analysis, possibly due to baseline values already being near normal ranges in study populations. Future clinical trials should address the limitations identified in the current work by establishing optimal dosing protocols (likely a 2000–8000 mg daily range based on current evidence), ensuring adequate intervention duration (minimum 8–12 weeks), and stratifying participants by baseline risk factors to identify the population most likely to benefit. Additionally, research exploring the mechanism behind Spirulina’s antihypertensive effects, particularly its ACE inhibitory peptides and nitric oxide production pathways, would enhance the understating of its potential therapeutic applications. Standardised reporting of outcomes would also facilitate more robust meta-analysis as the body of evidence continues to grow.

## Figures and Tables

**Table 1:** Characteristics of RCTs focusing on *Chlorella* supplementation.

Authors	Ref.	Year	Country	Study Design	Population	N Placebo	N Chlorella	Duration (Weeks)	Dosage (mg)	Age (Average)	Men (%)	BP	TC	LDL-C	HDL-C	TGs	Weight	BMI
Lee et al.	[17]	2010	South Korea	double-blinded, randomised, placebo-	Smokers	24	28	6	6300	37	48 (100%)	x						
Otsuki et al.	[18]	2011	Japan	blinded, randomised, crossover study design	Healthy	15	15	4	6000	20	30 (100%)						x	x
Kwak et al.	[19]	2012	South Korea	double-blinded, randomised, placebo-	Healthy	23	28	8	5000	35	20 (39%)	x	x	x	x	x	x	x
Otsuki et al.	[20]	2012	Japan	randomised controlled study	Healthy	10	10	4	6000	20	0 (0%)						x	
Miyazawa et al.	[21]	2013	Japan	double-blinded, randomised, placebo-	Healthy	6	6	8	8000	58	7 (58%)	x	x	x	x	x	x	x
Ryu et al.	[22]	2014	South Korea	double-blinded, randomised, placebo-	Mildly Hypercholeste	30	33	4	5000	50	19 (30%)		x	x	x	x		
Kim et al.	[23]	2016	South Korea	double-blinded, randomised, placebo-	Healthy	17	17	4	5000	24	4 (12%)		x	x	x			
Ebrahimi et al.	[24]	2017	Iran	double-blinded, randomised, placebo-	Obese	26	29	8	1200	35	30 (60%)						x	
Okada et al.	[25]	2018	Japan	double-blinded, parallel- arm controlled study	Healthy	13	14	4	7200	35	27 (100%)	x						
Chiu et al.	[26]	2021	Taiwan	double-blinded, randomised, placebo-	Healthy	23	21	12	27 mL ^a^	58	11 (25%)		x	x	x	x		
Hosseini et al.	[27]	2021	Iran	double-blinded, randomised, placebo-	Type 2 Diabetes	39	36	8	1500	56	29 (39%)	x	x	x	x	x	x	x
Sanayei et al.	[28]	2022	Iran	double-blinded, randomised, placebo-	Overweight/O bese	11	12	8	900	18–35 ^b^	0 (0%)						x	

Values are expressed as mean ± SD; Abbreviations: BP—blood pressure; TC—total cholesterol; LDL-C—low-density lipoprotein cholesterol; HDL-C—high-density lipoprotein cholesterol; TGs—tryglicerides; BMI—body mass index; ^a^ Chlorella Water Extract (CWE); ^b^ Only age range is reporter; x Biochemical parameter assessed.

**Table 2:** Characteristics of RCTs focusing on Spirulina supplementation.

Authors	Ref.	Year	Country	Study Design	Population	N Placebo	N Spirulina	Duration (Weeks)	Dosage (mg)	Age (Average)	Men (%)	BP	TC	LDL-C	HDL-C	TGs	FBG
Lee et al.	[29]	2008	South Korea	randomised controlled trial	Type 2 Diabetes Mellitus	18	19	12	8000	53	20 (54%)	x	x	x	x	x	x
Park et al.	[30]	2008	South Korea	double-blinded, randomised, placebo-	Healthy	37	41	16	8000	66	43 (34%)		x	x	x	x	
Jensen et al.	[31]	2016	USA	double-blinded, randomised, placebo-	Healthy	12	12	12	2000	46	5 (21%)	x					
Mickze et al.	[32]	2016	Poland	double-blinded, randomised, placebo-	Overweight and Hypertensive	20	20	12	2000	53	21 (53%)	x					
Szulinska et al.	[33]	2017	Poland	double-blinded, randomised, placebo-	Obese	25	25	12	2000	50	25 (50%)		x	x	x	x	
Zeinalian et al.	[34]	2017	Iran	double-blinded, randomised, placebo-	Obese	27	29	12	1000	34	9 (16%)		x	x	x	x	
Yousefi et al.	[35]	2018	Iran	randomised controlled study	Overweight/Obese	19	19	12	2000	40	7 (18%)		x	x	x	x	
Koite et al.	[36]	2022	France	double-blinded, randomised, placebo-	Metabolic Syndrome	20	20	12	200 ^a^	50	22 (55%)		x	x	x	x	x
Mohammad et al.	[37]	2022	Iran	single-blind and quasiexperimental	Overweight/Obese	15	15	8	1000	37	30 (100%)						x

Values are expressed as mean ± SD; Abbreviations: BP—blood pressure; TC—total cholesterol; LDL-C—ow-density lipoprotein cholesterol; HDL-C—high-density lipoprotein cholesterol; TGs—tryglicerides; FBG—fasting blood glucose; ^a^ liquid extract of Arthrospira (Spirulysat^®^); x Biochemical parameter assessed.

## Supplementary Materials

The following supporting information can be downloaded at https://www.mdpi.com/article/10.3390/nu17060943/s1. Section S1 contains forest plots summarising the differences in cardiometabolic indexes after treatment with *Chlorella* in relation to placebo; Section S2 contains forest plots summarising the differences in cardiometabolic indexes after treatment with Spirulina, in relation to placebo.
