# Supplementary material for: The Role of Chlorella and Spirulina as Adjuvants of Cardiovascular Risk Factor Control: A Systematic Review and Meta-Analysis of Randomised Controlled Trials
Source: Nutrients. 2025 Mar 7;17(6):943. doi: 10.3390/nu17060943 (PMC11945647; doi:10.3390/nu17060943)
Supplement: Supplementary file 1 [file nutrients-17-00943-s001.zip › nutrients-3507642-supplementary.pdf]

SUPPLEMENTARY MATERIALS

S.1. This supplementary material contains an assessment of the risk of bias and the forest plots summarising the differences in cardiometabolic indexes after treatment with *Chlorella* in relation to placebo.

Table S1. Bias risk assessment of *Chlorella* articles using NHLBI's assessment tool "Quality Assessment of Controlled Interventions Studies".

	Lee et al., 2010	Otsuki et al., 2011	Kwak et al., 2012	Otsuki et al., 2012	Miyazawa et al., 2013	Ryu et al., 2014	Kim et al., 2016	Ebrahimi et al., 2017	Okada et al., 2018	Chiu et al., 2021	Hosseini et al., 2021	Sanayei et al., 2022
Was the study described as randomized, a randomized trial, a randomized clinical trial, or an RCT?	yes	yes	yes	yes	yes	yes	yes	yes	yes	yes	yes	yes
Was the method of randomization adequate (i.e., use of randomly generated assignment)?	yes	yes	yes	NA	NA	yes	yes	yes	yes	yes	yes	yes
Was the treatment allocation concealed (so that assignments could not be predicted)?	yes	NA	yes	NA	NA	yes	yes	yes	yes	yes	yes	yes
Were study participants and providers blinded to treatment group assignment?	yes	yes	yes	yes	yes	yes	yes	yes	yes	yes	yes	yes
Were the people assessing the outcomes blinded to the participants' group assignments?	yes	NA	yes	no	NA	yes	yes	yes	yes	yes	yes	yes
Were the groups similar at baseline on important characteristics that could affect outcomes (e.g., demographics, risk factors, co-morbid conditions)?	yes	yes	yes	yes	yes	yes	yes	yes	yes	yes	yes	yes
Was the overall drop-out rate from the study at endpoint 20% or lower of the number allocated to treatment?	yes	NA	yes	NA	NA	yes	yes	no	NA	NA	yes	yes
Was the differential drop-out rate (between treatment groups) at endpoint 15 percentage points or lower?	yes	NA	yes	NA	NA	yes	yes	no	NA	NA	yes	yes
Was there high adherence to the intervention protocols for each treatment group?	NA	NA	NA	NA	NA	NA	yes	NA	NA	NA	yes	yes
Were other interventions avoided or similar in the groups (e.g., similar background treatments)?	yes	yes	yes	yes	yes	yes	yes	yes	yes	yes	yes	yes

Were outcomes assessed using valid and reliable measures, implemented consistently across all study participants?	yes	yes	yes	yes	yes	yes	yes	yes	yes	yes	yes	yes
Did the authors report that the sample size was sufficiently large to be able to detect a difference in the main outcome between groups with at least 80% power?	no	no	no	no	no	yes	yes	yes	no	no	yes	yes
Were outcomes reported or subgroups analyzed prespecified (i.e., identified before analyses were conducted)?	yes	yes	yes	yes	yes	yes	yes	yes	yes	yes	yes	yes
Were all randomized participants analyzed in the group to which they were originally assigned, i.e., did they use an intention-to-treat analysis?	yes	yes	yes	yes	yes	yes	yes	yes	yes	yes	yes	yes
Quality Rating	Good	Good	Good	Good	Good	Good	Good	Good	Good	Good	Good	Good

Study or Subgroup	Chlorella Mean	SD	Total	Control Mean	SD	Total	Weight	Std. mean difference IV, Random, 95% CI
Hosseini et al., 2021	-0.61	18.13	36	0	24.48	39	34.7%	-0.03 [-0.48 , 0.43]
Kwak et al., 2012	-0.4	19.64	23	-0.32	17.63	28	23.4%	-0.00 [-0.56 , 0.55]
Lee et al., 2010	-0.9	13.55	28	-4	24.21	24	23.9%	0.16 [-0.39 , 0.71]
Miyazawa et al., 2013	-1.7	36.41	6	-1.7	16.51	6	5.6%	0.00 [-1.13 , 1.13]
Okada et al., 2018	-1.9	23.54	14	3.2	21.74	13	12.4%	-0.22 [-0.98 , 0.54]
**Total (95% CI)**			**107**			**110**	**100.0%**	**0.00 [-0.27 , 0.27]**

Heterogeneity: Tau² = 0.00; Chi² = 0.66, df = 4 (P = 0.96); I² = 0%
Test for overall effect: Z = 0.00 (P = 1.00)
Test for subgroup differences: Not applicable

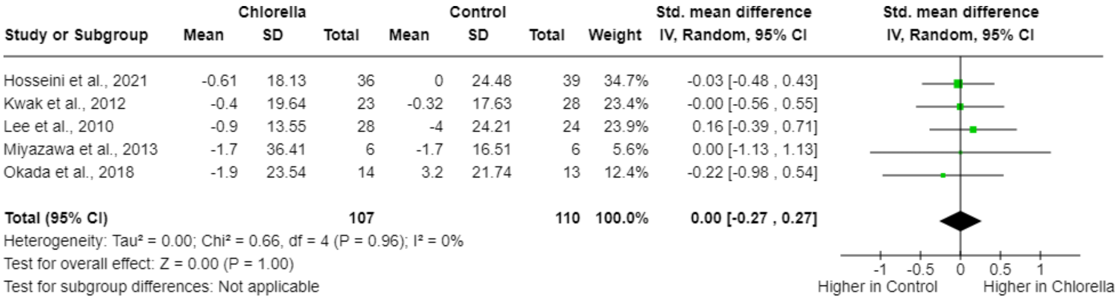

Figure S1. Delta analysis of systolic blood pressure (SBP) in *Chlorella* and placebo groups.

Study or Subgroup	Chlorella Mean	SD	Total	Placebo Mean	SD	Total	Weight	Std. mean difference IV, Random, 95% CI
Hosseini et al. 2021	0.08	13.87	36	-0.28	15.29	39	34.9%	0.02 [-0.43 , 0.48]
Kwak et al. 2012	0	13.91	23	-3	15.79	28	23.4%	0.20 [-0.36 , 0.75]
Lee et al. 2010	1.5	7.94	28	1.2	15.13	24	24.1%	0.03 [-0.52 , 0.57]
Miyazawa et al. 2013	-1.1	23.43	6	-1.7	14.37	6	5.6%	0.03 [-1.10 , 1.16]
Okada et al. 2018	-6.3	12.38	14	1.6	14	13	12.0%	-0.58 [-1.35 , 0.19]
**Total**			**107**			**110**	**100.0%**	**-0.01 [-0.27 , 0.26]**

Test for overall effect: Z = 0.05 (P = 0.96)
Test for subgroup differences: Not applicable
Heterogeneity: Tau² = 0.00; Chi² = 2.68, df = 4 (P = 0.61); I² = 0%

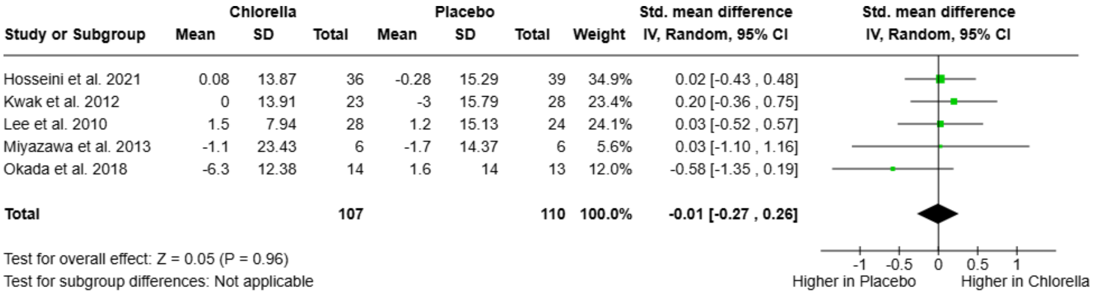

Figure S2. Baseline diastolic blood pressure in the *Chlorella* and placebo groups.

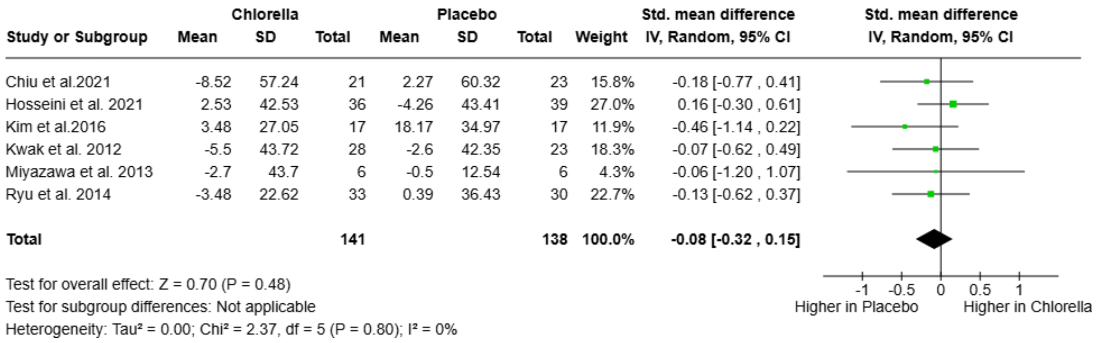

Figure S3. Delta analysis of total cholesterol (TC) in *Chlorella* and placebo groups.

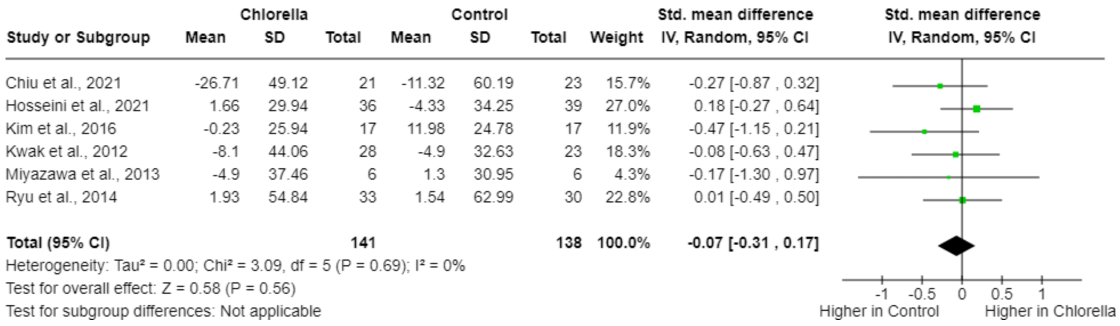

Figure S4. Delta analysis of low-density lipoprotein cholesterol (LDL-C) in *Chlorella* and placebo groups.

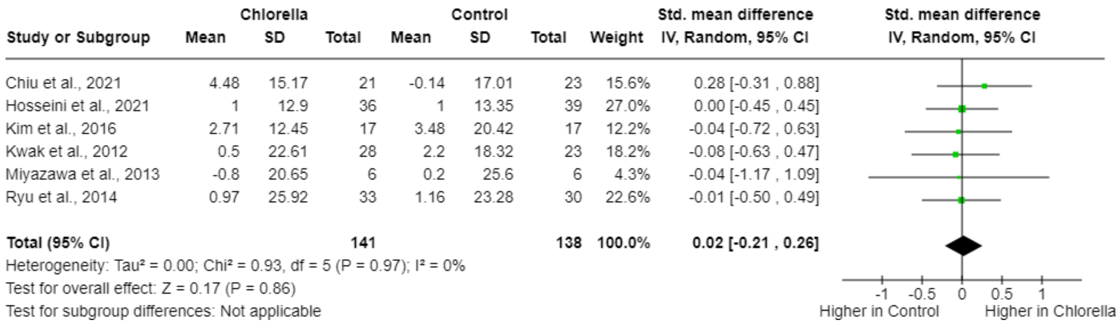

Figure S5. Delta analysis of high-density lipoprotein cholesterol (HDL-C) in *chlorella* and placebo groups.

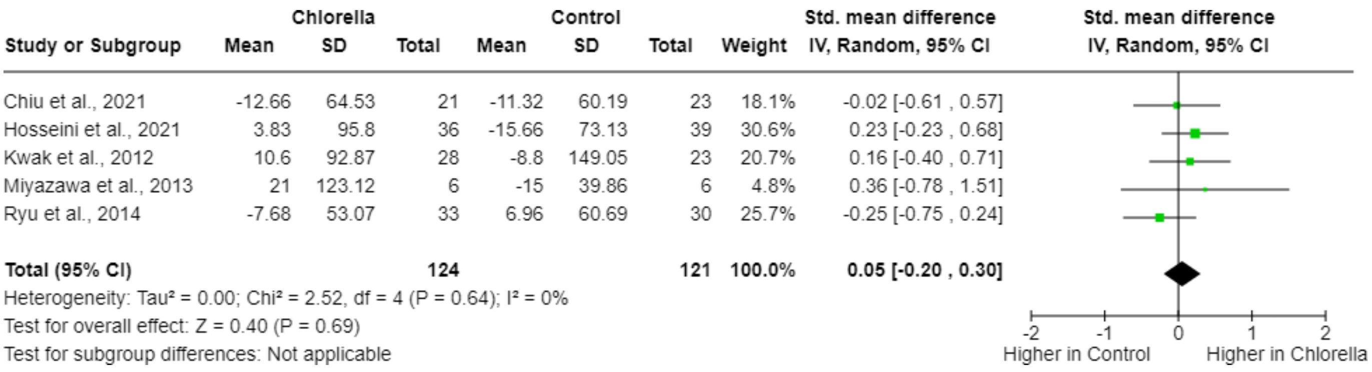

Figure S6. Delta analysis of triglyceride (TG) in *Chlorella* and placebo groups.

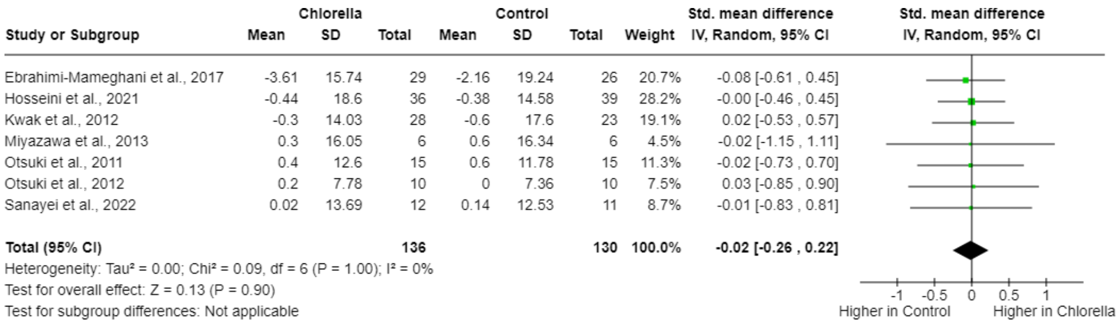

Figure S7. Delta analysis of weight in *Chlorella* and placebo groups.

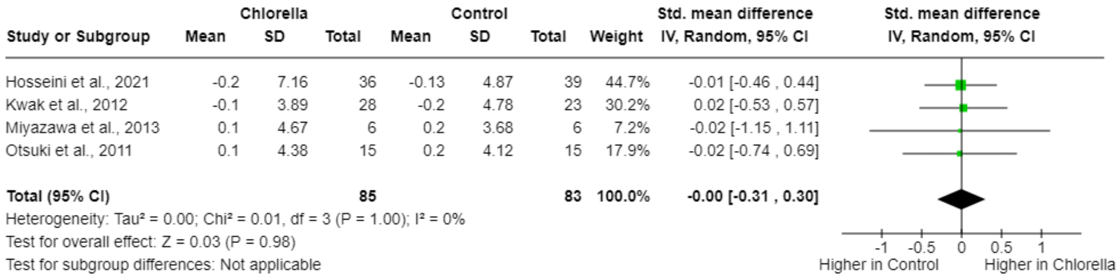

Figure S8. Delta analysis of body mass index (BMI) in *chlorella* and placebo groups.

S.2. This supplementary material contains an assessment of the risk of bias and forest plots summarising the differences in cardiometabolic indexes after treatment with Spirulina in relation to placebo.

Table S2. Bias risk assessment of Spirulina articles using NHLBI's assessment tool "Quality Assessment of Controlled Interventions Studies"

	Lee et al., 2008	Park et al., 2008	Jensen et al., 2015	Mickze et al., 2016	Szulinska et al., 2017	Zeinalian et al., 2017	Yousefi et al., 2018	Koite et al., 2022	Mohammad et al., 2022
Was the study described as randomized, a randomized trial, a randomized clinical trial, or an RCT?	yes	yes	yes	yes	yes	yes	yes	yes	no
Was the method of randomization adequate (i.e., use of randomly generated assignment)?	yes	NA	NA	yes	yes	yes	yes	NA	yes
Was the treatment allocation concealed (so that assignments could not be predicted)?	NA	yes	yes	yes	yes	yes	yes	yes	no
Were study participants and providers blinded to treatment group assignment?	yes	yes	yes	yes	yes	yes	yes	yes	no
Were the people assessing the outcomes blinded to the participants' group assignments?	NA	yes	yes	yes	yes	yes	yes	yes	no

Were the groups similar at baseline on important characteristics that could affect outcomes (e.g., demographics, risk factors, co-morbid conditions)?	yes	yes	yes	yes	yes	yes	yes	yes	yes
Was the overall drop-out rate from the study at endpoint 20% or lower of the number allocated to treatment?	NA	NA	NA	NA	yes	yes	yes	yes	NA
Was the differential drop-out rate (between treatment groups) at endpoint 15 percentage points or lower?	NA	NA	NA	NA	yes	NA	NA	yes	NA
Was there high adherence to the intervention protocols for each treatment group?	NA	NA	yes	yes	yes	NA	yes	yes	NA
Were other interventions avoided or similar in the groups (e.g., similar background treatments)?	yes	yes	yes	yes	yes	yes	yes	yes	yes
Were outcomes assessed using valid and reliable measures, implemented consistently across all study participants?	yes	yes	yes	yes	yes	yes	yes	yes	yes
Did the authors report that the sample size was sufficiently large to be able to detect a difference in the main outcome between groups with at least 80% power?	no	no	no	yes	no	yes	no	yes	no
Were outcomes reported or subgroups analyzed prespecified (i.e., identified before analyses were conducted)?	yes	yes	yes	yes	yes	yes	yes	yes	yes
Were all randomized participants analyzed in the group to which they were originally assigned, i.e., did they use an intention-to-treat analysis?	yes	yes	yes	yes	yes	yes	yes	yes	yes
Quality rating	Good	Good	Good	Good	Good	Good	Good	Good	Good

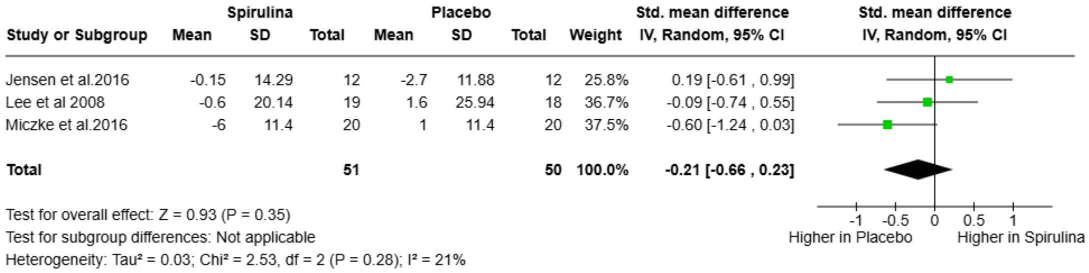

Figure S9. Delta analysis of systolic blood pressure (SBP) in Spirulina and placebo groups.

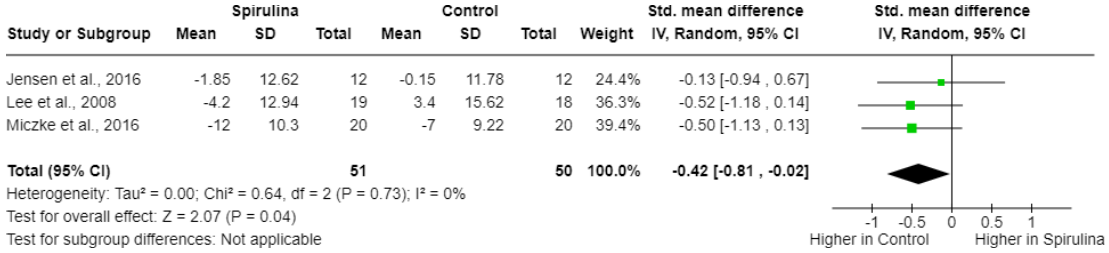

Figure S10. Delta analysis of diastolic blood pressure (DBP) in Spirulina and placebo groups.

Study or Subgroup	Spirulina Mean	Spirulina SD	Spirulina Total	Control Mean	Control SD	Control Total	Weight	Std. mean difference IV, Random, 95% CI
Koite et al., 2022	-3	49.52	20	-5	62.97	20	13.5%	0.03 [-0.59 , 0.65]
Lee et al., 2008	0.7	40.52	19	10.3	52.9	18	12.4%	-0.20 [-0.85 , 0.45]
Park et al., 2008	-9.79	70.76	41	1.17	75.4	37	26.1%	-0.15 [-0.59 , 0.30]
Szulinska et al., 2017	-11.31	54.87	25	7.57	46.57	25	16.5%	-0.37 [-0.92 , 0.19]
Yousefi et al., 2018	-7.37	59.7	19	2.32	56.41	19	12.7%	-0.16 [-0.80 , 0.47]
Zeinalian et al., 2017	-10.38	47.03	29	-4.22	39.02	27	18.8%	-0.14 [-0.66 , 0.38]
**Total (95% CI)**			**153**			**146**	**100.0%**	**-0.17 [-0.39 , 0.06]**

Heterogeneity: $Tau^2 = 0.00$; $Chi^2 = 0.92$, df = 5 (P = 0.97); $I^2 = 0\%$
Test for overall effect: Z = 1.43 (P = 0.15)
Test for subgroup differences: Not applicable

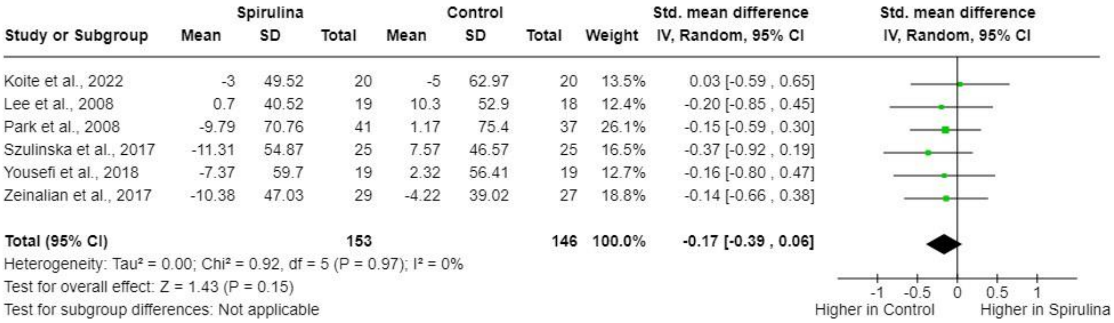

Figure S11. Delta analysis of total cholesterol (TC) in Spirulina and placebo groups.

Study or Subgroup	Spirulina Mean	Spirulina SD	Spirulina Total	Control Mean	Control SD	Control Total	Weight	Std. mean difference IV, Random, 95% CI
Koite et al., 2022	-3	46.67	33	-6	50.33	20	16.2%	0.06 [-0.49 , 0.62]
Lee et al., 2008	2.6	40.54	19	5.9	39.3	18	12.0%	-0.08 [-0.73 , 0.56]
Park et al., 2008	-5.76	75.36	41	-0.65	83.73	37	25.3%	-0.06 [-0.51 , 0.38]
Szulinska et al., 2017	-19.35	41.85	25	0	49.26	25	15.9%	-0.42 [-0.98 , 0.14]
Yousefi et al., 2018	-9.05	35.65	19	-2.74	32.57	19	12.3%	-0.18 [-0.82 , 0.46]
Zeinalian et al., 2017	-0.85	45.04	29	-3.22	30.41	27	18.2%	0.06 [-0.46 , 0.58]
**Total (95% CI)**			**166**			**146**	**100.0%**	**-0.09 [-0.32 , 0.13]**

Heterogeneity: $Tau^2 = 0.00$; $Chi^2 = 2.00$, df = 5 (P = 0.85); $I^2 = 0\%$
Test for overall effect: Z = 0.82 (P = 0.41)
Test for subgroup differences: Not applicable

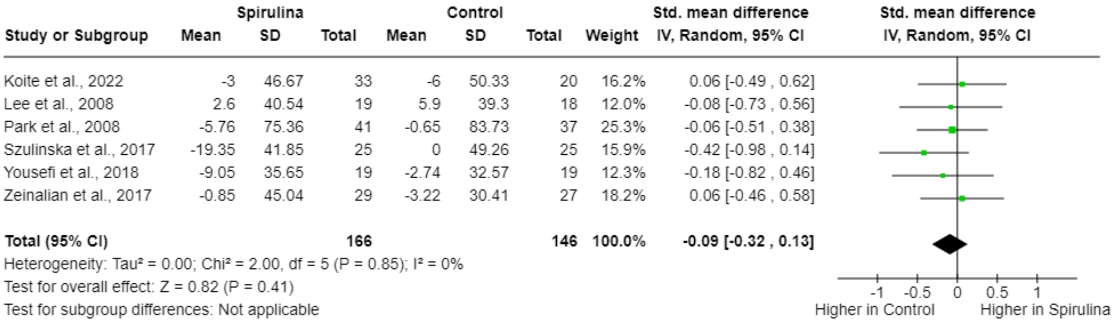

Figure S12. Delta analysis of low-density lipoprotein cholesterol (LDL-C) in Spirulina and placebo groups.

Study or Subgroup	Spirulina Mean	Spirulina SD	Spirulina Total	Control Mean	Control SD	Control Total	Weight	Std. mean difference IV, Random, 95% CI
Koite et al., 2022	1	16.64	20	1	22.2	20	14.7%	0.00 [-0.62 , 0.62]
Lee et al., 2008	1.3	17.3	19	0.5	20.44	18	13.6%	0.04 [-0.60 , 0.69]
Park et al., 2008	-2.05	26.9	41	1.31	28.69	19	19.0%	-0.12 [-0.67 , 0.42]
Szulinska et al., 2017	0	16.4	25	-3.93	21.88	25	18.3%	0.20 [-0.36 , 0.76]
Yousefi et al., 2018	-0.95	14.74	19	-0.69	10.9	19	13.9%	-0.02 [-0.66 , 0.62]
Zeinalian et al., 2017	2.2	13.51	29	3.49	14.92	27	20.5%	-0.09 [-0.61 , 0.43]
**Total (95% CI)**			**153**			**128**	**100.0%**	**-0.00 [-0.24 , 0.24]**

Heterogeneity: $Tau^2 = 0.00$; $Chi^2 = 0.82$, df = 5 (P = 0.98); $I^2 = 0\%$
Test for overall effect: Z = 0.02 (P = 0.99)
Test for subgroup differences: Not applicable

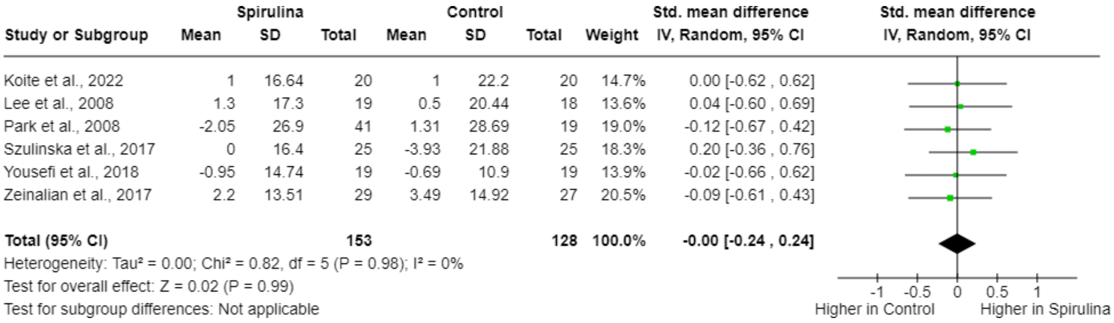

Figure S13. Delta analysis of high-density lipoprotein cholesterol (HDL-C) in Spirulina and placebo groups.

Study or Subgroup	Spirulina Mean	Spirulina SD	Spirulina Total	Control Mean	Control SD	Control Total	Weight	Std. mean difference IV, Random, 95% CI
Koite et al., 2022	-11	73.25	20	14	114.56	20	13.4%	-0.25 [-0.88 , 0.37]
Lee et al., 2008	-27.3	79.58	19	17.5	97.35	18	12.0%	-0.49 [-1.15 , 0.16]
Park et al., 2008	-4.19	160.57	41	2.09	119.75	37	26.2%	-0.04 [-0.49 , 0.40]
Szulinska et al., 2017	-9.35	119.34	25	9.35	144.46	25	16.8%	-0.14 [-0.69 , 0.42]
Yousefi et al., 2018	7.33	150.69	19	10.33	164.6	19	12.8%	-0.02 [-0.65 , 0.62]
Zeinalian et al., 2017	-7.48	81.42	29	-15.26	107.39	27	18.8%	0.08 [-0.44 , 0.61]
**Total (95% CI)**			**153**			**146**	**100.0%**	**-0.12 [-0.34 , 0.11]**

Heterogeneity: $Tau^2 = 0.00$; $Chi^2 = 2.21$, df = 5 (P = 0.82); $I^2 = 0\%$
Test for overall effect: Z = 0.99 (P = 0.32)
Test for subgroup differences: Not applicable

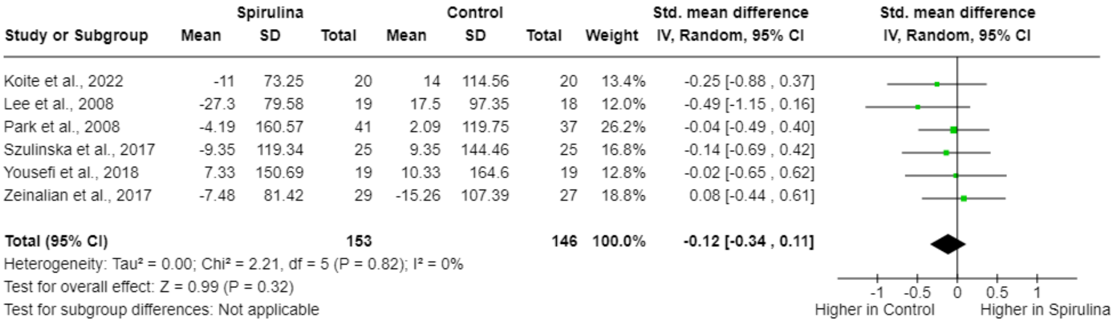

Figure S14. Delta analysis of triglyceride (TG) in Spirulina and placebo groups.

Study or Subgroup	Spirulina Mean	Spirulina SD	Spirulina Total	Control Mean	Control SD	Control Total	Weight	Std. mean difference IV, Random, 95% CI
Koite et al., 2022	6.1	15.76	20	2.1	17.02	20	37.3%	0.24 [-0.38 , 0.86]
Lee et al., 2008	2.3	24.36	19	2.1	37.39	18	34.7%	0.01 [-0.64 , 0.65]
Mohammad et al., 2022	-2.14	9.83	15	-0.53	7.05	15	28.0%	-0.18 [-0.90 , 0.53]
**Total (95% CI)**			**54**			**53**	**100.0%**	**0.04 [-0.34 , 0.42]**

Heterogeneity: $Tau^2 = 0.00$; $Chi^2 = 0.78$, df = 2 (P = 0.68); $I^2 = 0\%$
Test for overall effect: Z = 0.21 (P = 0.84)
Test for subgroup differences: Not applicable

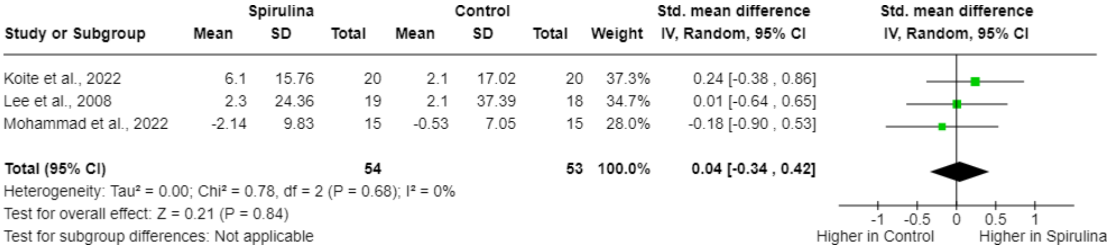

Figure S15. Delta analysis of fasting blood glucose (FBG) in Spirulina and placebo groups.
